# Analysis of Various Pickup Coil Designs in Nonmodule-Type GaN Power Semiconductors

**DOI:** 10.3390/s20216066

**Published:** 2020-10-25

**Authors:** Ui-Jin Kim, Rae-Young Kim

**Affiliations:** The Department of Electrical and Biomedical Engineering, Hanyang University, Seoul 04763, Korea; anan574432@hanyang.ac.kr

**Keywords:** GaN, Rogowski coil, pick-up coil, double pulse test, switch current

## Abstract

Gallium nitride (GaN) devices are advantageous over conventional Silicon (Si) devices in terms of their small size, low on-resistance, and high dv/dt characteristics; these ensure a high integrated density circuit configuration, high efficiency, and fast switching speed. Therefore, in the diagnosis and protection of a system containing a GaN power semiconductor, the transient state for accurate switch current measurement must be analyzed. The pick-up coil, as a current sensor for switch current measurement in a system comprising a surface-mount-device-type nonmodular GaN power semiconductor, has the advantages of a higher degree-of-freedom configuration for its printed circuit board, a relatively small size, and lower cost than other current sensors. However, owing to the fast switching characteristics of the GaN device, a bandwidth of hundreds MHz must be secured along with a coil configuration that must overcome the limitations of relatively low sensitivity of the conventional current sensor. This paper analyzes the pick-up coil sensor models that can achieve optimal bandwidth and sensitivity for switch current measurement in GaN based device. So four configurable pick-up coil models are considered and compared according to coil-parameter using mathematical methods, magnetic, and frequency-response analysis. Finally, an optimal coil model is proposed and validated using a double-pulse test.

## 1. Introduction

A power semiconductor with a wideband gap (WBG) is a device capable of high-density integration owing to its high energy efficiency and high-speed switching and has the advantage of a small size with strong dv/dt characteristics at a high temperature compared to a conventional Si device. Among the WBG semiconductors, GaN devices have lower stability to high temperatures than Silicon carbide devices but can be switched faster based on high dv/dt characteristics. Recently, studies have been conducted to improve thermal stability based on technological advances [1,2,3,4].

However, the high dv/dt characteristic due to the fast switching speed of GaN is highly sensitive to noise and inductance [5,6]. Therefore, the problems of peak values and pulsation components due to noise and loop inductance components in the switching transient periods must be overcome to achieve a stable device operation [1,5]. In addition, to obtain accurate transient response characteristics for measuring the switching current and protecting the system, the bandwidth of the sensor must be sufficiently guaranteed [4]. Current sensors for integrated systems have been extensively studied for improving sensor performance based on evaluation criteria such as size, cost, transient response characteristics, noise immunity, and sufficient bandwidth. Existing current sensors for measuring the switch current include methods using Ohm’s law of resistance, a magnetic field sensor, and Faraday’s induction law. The most commonly used device is the shunt resistor, which is a representative sensor using Ohm’s law of resistance, relatively inexpensive, and has a long commercialization history [7,8,9]. However, if the current sensor is used in a printed circuit board (PCB) for system protection, its configuration as a built-in type sensor is difficult and the loop inductance value increases with the installation of a shunt resistor; these disadvantages must be overcome. Magneto-resistor sensors [10,11,12,13,14] and Hall-effect sensors [15,16,17,18,19,20] are isolated static current sensors used in magnetic field sensors and have the advantages of DC and AC measurement [11,12].

Current transformers (CT) [21,22,23,24] and Rogowski coils [25,26,27,28,29,30,31,32] are the magnetic field induction based transducer by the current passing through the closed area of the coil according to Faraday’s law. These sensors, as an isolated sensor, could be used to conduct wide-scale current measurements in a relatively high bandwidth and at a low price [26,27,28,29]. Compared to the CT, the Rogowski coil portrays a lower core loss and a higher linear characteristic owing to its non-saturation characteristic, regardless of the current range, by using an air core instead of a ferromagnetic core [22,30]. However, fast-switching-power semiconductors, such as GaN, are sensitive to noise and loop inductance due to high dv/dt, and therefore sufficient bandwidth is required to increase the transient response characteristics. In addition, it experiences the problem of relatively low sensitivity due to the nature of the air core [33,34,35,36,37]. Accordingly, to design a coil, an appropriate number of coil turns must be determined and a new coil design must be developed. The toroidal-form winding coil is the most common Rogowski coil [37] but has many design limitations when built in a PCB for measurement of switching current and system protection. Therefore, the toroidal form of the Rogowski coil can only be configured externally by using a modular power semiconductor rather than a PCB built-in semiconductor [32,38,39]. This is because the windings of the toroidal coil require the conductor, through which the measured current flows, to be wrapped around the core. However, if the conductor is in its trace form, it is difficult to design the coil in the form of a built-in PCB.

The pick-up coil is a sensor capable of a built-in PCB and was proposed in 2011, with the goal of decreasing the size and increasing the bandwidth [40]. Similar to the existing Rogowski coil, the operating principle of the pick-up coil involves the measurement of the induced output voltage of the coil by receiving a part of the magnetic field flux generated by the measured current in the coil according to Faraday’s law. Compared to the existing toroidal-type Rogowski coil, the structure of the pick-up coil is designed with a higher degree of freedom because its structure can attract a portion of the magnetic field flux and not the entire magnetic field of radiation in all directions (360°) generated from the measured current. With such a structure, the pickup coil can be configured as a PCB built-in type in a nonmodular power semiconductor as well as a modular power semiconductor. However, the reduction in measurement sensitivity due to the significantly reduced mutual inductance value compared to the toroidal type is unavoidable [40,41,42].

In this study, we analyzed the parameters for the optimum configuration of the pick-up coil that can measure the switch current in a half-bridge model composed of a nonmodular GaN power semiconductor. In addition, we propose a structure that can simultaneously increase the bandwidth and sensitivity of a device by comparing and analyzing the frequency response and magnetic flux flow of four configurable pickup coils.

This paper is organized as follows. Section 2 presents the basic structure and composition of the four pick-up coil models that include an integrator that can be built into the PCB circuit. Section 3 presents the analyses of the mutual inductance, frequency response, and magnetic properties of the coil models. Section 4 presents the experimental verification of the method used in this study and Section 5 draws the conclusion.

## 2. Design of the Current Sensor

### 2.1. Pick-Up Coil Design

**Figure 1:**
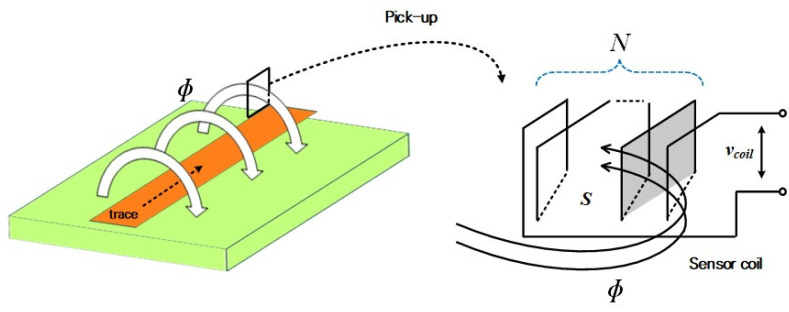
Schematic of the pick-up coil.

Figure 1 shows the schematic of measuring the current through the built-in PCB pick-up coil when the switch current flows in the PCB trace. According to Faraday’s law, a part of the magnetic-field flux, *ф*, generated by the primary current flowing in the trace enters the coil and an electromotive force (EMF) is induced at the terminal of the coil. The induced EMF of the coil is represented by the rate of change of time of the magnetic-field flux, and it presents negative values according to Lenz’s law. The induced EMF is proportional to mutual inductance value *M* between the coil and measurement trace. The mutual inductance value shown in Equation(2) is proportional to the number of turns of the sensor coil in Figure 1 and is proportional to the area where the magnetic-field flux is incident [25,26]:

(1) $$e\left(t\right)=-\frac{d\phi }{dt}=-M\frac{di_{1}}{dt},$$

(2) $$M=\mu_{o}\cdot N\cdot S,$$

**Figure 2:**
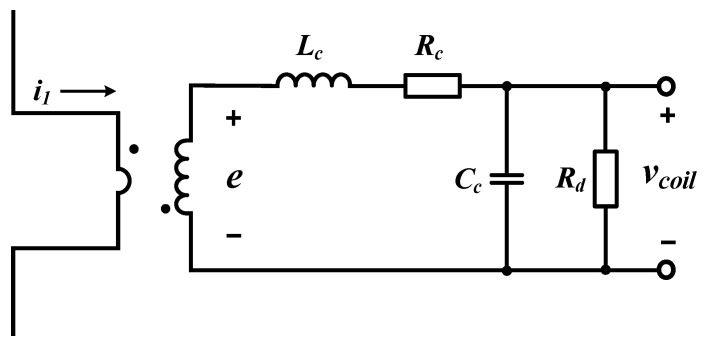
Equivalent lumped circuit of the pick-up coil.

Figure 2 shows that the circuit for the pick-up coil can be equivalent to that of the sensor coil based on the lumped model theory. According to the lumped circuit, the transfer function of the coil’s output to the measured current is represented as:

(3) $$G_{coil}(s)=\frac{V_{coil}(s)}{I_{1}(s)}=\frac{M\cdot s}{(L_{c}C_{c})s^{2}+s(\frac{L_{c}+R_{c}R_{d}C_{c}}{R_{d}})+\frac{R_{c}+R_{d}}{R_{d}}},$$

where *L_c_* is the magnetization inductance of the coil, *R_c_* is the self-resistance value of the coil, *C_c_* is the self-capacitance value of the coil, and *M* denotes the mutual inductance between the coil and conductor.

Here, optimal damping resistance *R_d_* is the same as Equation (5) based on the damping ratio *ζ* [26,30]:

(4) $$\varsigma =\frac{1}{2\sqrt{L_{c}C_{c}}}\left(\frac{L_{c}}{R_{d}}+R_{c}C_{c}\right)\sqrt{\frac{R_{d}}{R_{d}+R_{c}}},$$

(5) $$R_{d}=\frac{1}{2\zeta }\sqrt{\frac{L_{c}}{C_{c}}}.$$

**Figure 3:**
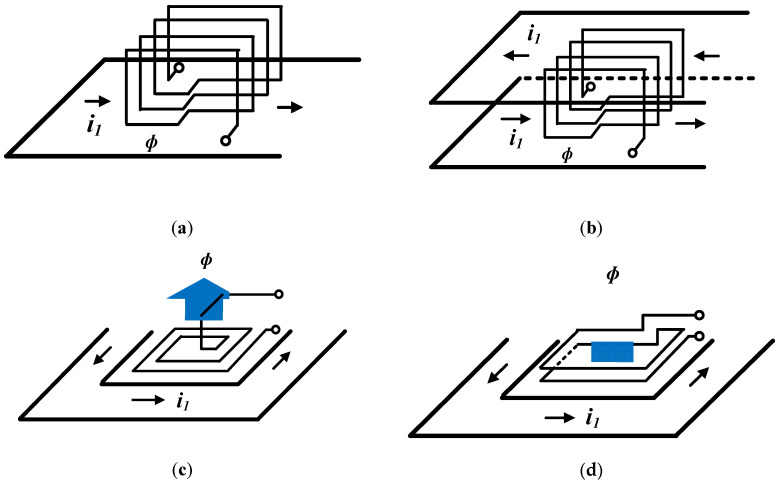
Magnetic-field flow through a (**a**) model-1: pick-up coil with conductor trace on one side; (**b**) model-2: pick-up coil with conductor trace on two sides; (**c**) model-3: spiral coil with conductor trace on three sides and (**d**) model-4: rectangular coil with conductor trace on three sides.

Figure 3 depicts four pick-up coil models that can be configured using the built-in PCB, and the arrows show the direction of the magnetic-field flux generated by the primary current. Model-1 and model-2 show models consisting of a vertical layer of the current trace and pick-up coil within a limited area on the PCB. Figure 3a shows the case in which the current conductor trace is located in the direction perpendicular to the plane, where the magnetic-field flux in the coil is incident at one side, and Figure 3b shows the coil structure in the case that the conductor is located on two sides. As shown in Figure 3b, the conductor has more magnetic flux entering the coil in the same direction than in the case of only one conductor trace [Figure 3a] because the measured current flows through the both upper and lower trace surfaces. Therefore, model-2 shows a higher value of mutual inductance between the conductor and coil compared to model-1, according to Equation (1). In both cases, the magnetization and mutual inductance values were determined according to the number of coil turns.

Model-3 and model-4 show the conductor traces through which the measured current flows wraps the sensor coil in three planes on the same plane; the magnetic line generated by the current on the three sides enters the sensor. In addition, the conductor trace, through which the measured current flows, wraps the sensor coil on the three sides on the same plane and the magnetic-field flux generated by the current at the three sides enters the sensor coil in the same direction. While the model-3 is composed of a single-layer spiral coil, the configuration in Figure 3d shows a 2-layer turn rectangular-coil structure. In these case, Compared to the model-1 and model-2, the direction of the generated magnetic-field flux is in the vertical direction rather than in the horizontal direction.

### 2.2. Sensor Design with Active Integration

**Figure 4:**
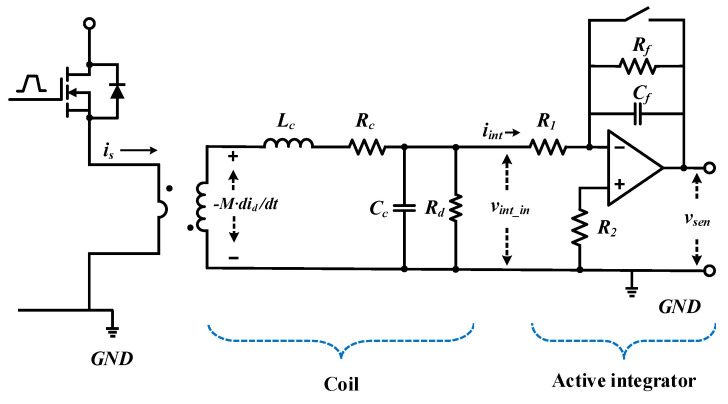
Comprehensive equivalent circuit sensor.

The entire sensor consists of a coil and an integrator, and the overall equivalent circuit of the sensor is shown in Figure 4. The integrator consists of an active inverting integrator, and the resistor *R_f_* value is selected and placed parallel to integrator capacitor *C_f_* to limit the infinite gain value of the inverting input stage appearing in a low-frequency environment to a finite gain value [43]. Here, the transfer function of the sensor output versus the integrator input is represented as

(6) $$\frac{V_{sen}}{V_{int\_in}}=\left[-\frac{R_{f}}{R_{1}}\right]\cdot \left[\frac{1}{1+j\omega R_{f}C_{f}}\right].$$

**Figure 5:**
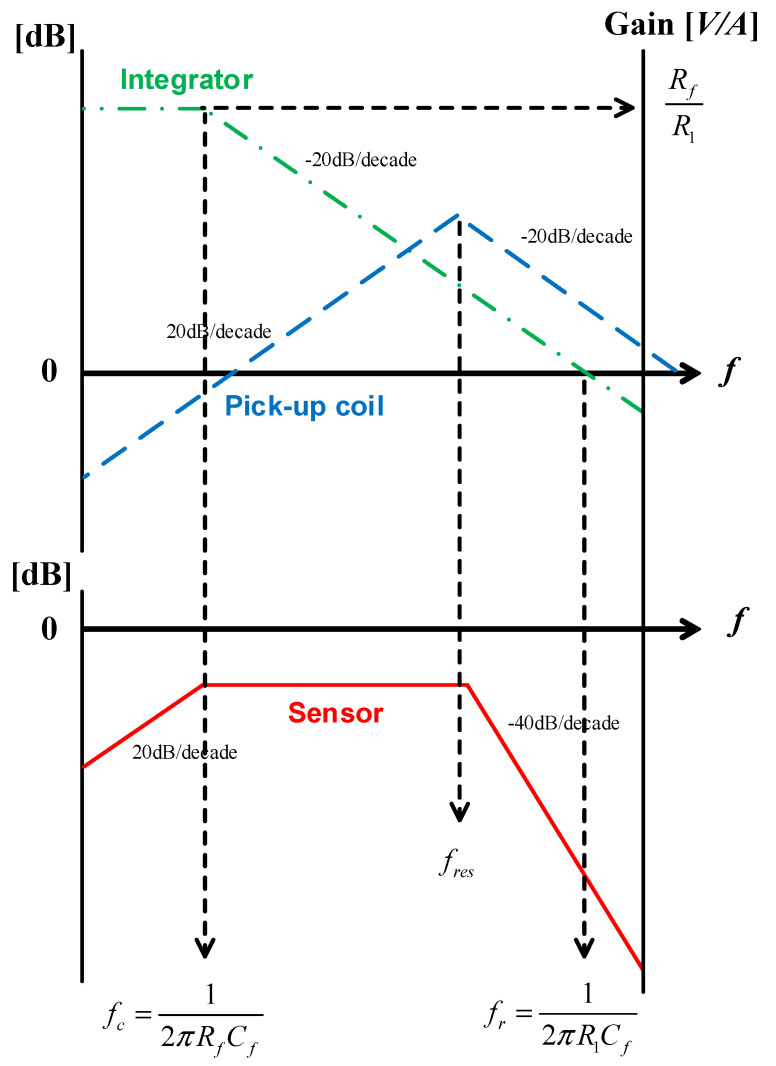
Frequency characteristics of a sensor with an active integrator.

In Figure 5, in the magnitude frequency response of the sensor, the unit gain of frequency, *f_r_*, is calculated using Equation (9) according to the gain bandwidth product; the graph shows a −40-dB/decade slope at the corner frequency value of the coil.

(7) $$f_{c}=\frac{1}{2\pi R_{f}C_{f}}$$

(8) $$f_{r}=gain_{f\to f_{c}}\cdot f_{c}=\frac{1}{2\pi R_{1}C_{f}}$$

For all sensors, including coils and integrators, the transfer function of the sensor output versus primary current is calculated as.

(9) $$G_{sen}(s)=\frac{V_{sen}(s)}{I_{s}(s)}=\left[-\frac{R_{f}}{R_{1}}\right]\left[\frac{1}{1+R_{f}\cdot Cs}\right]\times \frac{M\cdot s}{(L_{c}C_{c})s^{2}+s(\frac{L_{c}+R_{c}R_{d}C_{c}}{R_{d}})+\frac{R_{c}+R_{d}}{R_{d}}}.$$

## 3. Analysis of Current Sensors

Table 1 shows the results of parameter analysis for inductance, resistance, and bandwidth for the four coil models. At this time, Maxwell and Q3D, a specialized tool for finite element analysis, were used to analyze parameters for inductance and resistance values. And for comparison between sensors with specific sensitivity, we compared each other based on the same mutual inductance value of 2.8 nH. So, in the case of model-3 and model-4 in Table 1, it is possible to configure a multi-turn coil on a multi-layer according to the purpose of use when designing an actual coil. In the case of bandwidth information, it is the result of frequency response analysis using MATLAB based on a given coil parameter value.

### 3.1. Mutual Inductance

**Figure 6:**
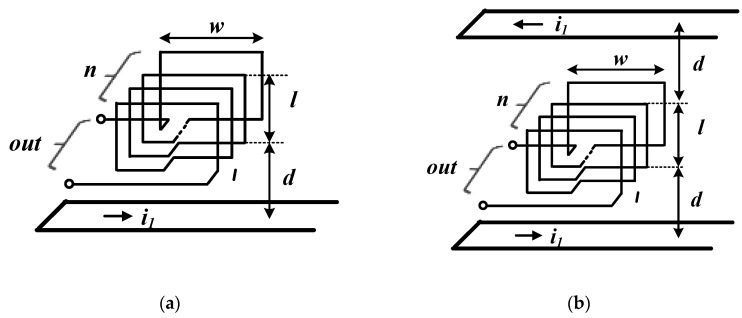

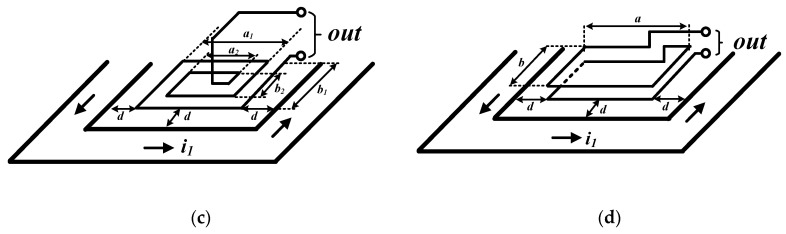
Geometry of the (**a**) model-1; (**b**) model-2; (**c**) model-3 and (**d**) model-4.

The amount of the magnetic flux line formed by the primary current is represented as the value of the magnetic flux density passing through the incident surfaces of pick-up coil. In addition, the mutual inductance between the conductor and coil is determined based on the EMF value of the formed coil. Here, *d* is the distance between the trace and coil through which the primary current flows, *s* is the incident surface in the magnetic-field flux to the coil, *l* is the height of the coil’s cross section, *w* is the width of the coil, and *n* is the number of coil turns. In the case of a pick-up coil composed of a conductor trace on one side, as shown in Figure 6a, the magnetic-field flux generated by the primary current is represented as

(10) $$\phi =\int_{s}Bds=\frac{\mu_{o}\cdot I}{2\pi }\int_{d}^{l+d}\frac{1}{y}\cdot w\cdot dy=\frac{\mu_{o}\cdot w\cdot I}{2\pi }\ln\frac{\left(l+d\right)}{d}.$$

Here, the EMF of the coil based on the magnetic-field flux in the core is the same as that in the *n*-turn coil configuration, i.e.,

(11) $$e(t)=-\frac{n\cdot d\phi }{dt}=-\frac{n\cdot \mu_{o}\cdot w}{2\pi }\ln\frac{\left(l+d\right)}{d}\frac{di}{dt}.$$

From Equations (1), (2), and (11), mutual inductance *M* is calculated as

(12) $$M=\frac{n\cdot \mu_{o}\cdot w}{2\pi }\ln\frac{\left(l+d\right)}{d}.$$

In the case the pick-up coil is inserted between the conductor traces on two sides, as shown in Figure 6b, the magnetic-field flux based on the primary current flowing at the top and bottom of the trace enters the coil in the same direction. Compared to the previous case, the coil comprises twice the magnetic flux, and mutual inductance *M* is calculated as shown in Equation (15):

(13) $$\phi =\int_{s}Bds=\frac{\mu_{o}\cdot I}{\pi }\int_{d}^{l+d}\frac{1}{y}\cdot w\cdot dy=\frac{\mu_{o}\cdot w\cdot I}{\pi }\ln\frac{\left(l+d\right)}{d},$$

(14) $$M=\frac{n\cdot \mu_{o}\cdot w}{\pi }\ln\frac{\left(l+d\right)}{d}.$$

Figure 6c shows the coil model in which the conductor traces are located on the three sides surrounding the spiral pick-up coil on the same plane. The magnetic-field flux generated by the current flowing through each trace is incident into the coils in the same direction. When the distance between two adjacent coil lines in the spiral coil is c, the magnetic flux incident inside the coil in the spiral coil composed of m-turns is as follows.

(15) $$\phi =\frac{\mu_{o}\cdot I}{\pi }\sum_{i=1}^{m}\left\{2b_{1}\ln\left(\frac{a_{1}+d+(i-1)\cdot c}{d+(i-1)\cdot c}\right)+a_{1}\ln\left(\frac{b_{1}+d+(i-1)\cdot c}{d+(i-1)\cdot c}\right)\right\}.$$

And, the mutual inductance between a coil composed of a single layer and a conductor trace is calculated using Equation (17):

(16) $$e(t)=\frac{\mu_{o}}{\pi }\sum_{i=1}^{m}\left[\left\{2b_{i}\ln\left(\frac{a_{i}+d+\left(i-1\right)c}{d+\left(i-1\right)c}\right)\right\}+\left\{a_{i}\ln\left(\frac{b_{i}+d+\left(i-1\right)c}{d+\left(i-1\right)c}\right)\right\}\right]\frac{di}{dt},$$

(17) $$M=\frac{\mu_{o}}{\pi }\sum_{i=1}^{m}\left[\left\{2b_{i}\ln\left(\frac{a_{i}+d+\left(i-1\right)c}{d+\left(i-1\right)c}\right)\right\}+\left\{a_{i}\ln\left(\frac{b_{i}+d+\left(i-1\right)c}{d+\left(i-1\right)c}\right)\right\}\right].$$

And, the mutual inductance of the coil composed of m-turns on the n-layer is as follows.

(18) $$M_{n}=\frac{\mu_{o}\cdot n}{\pi }\sum_{i=1}^{m}\left[\left\{2b_{i}\ln\left(\frac{a_{i}+d+\left(i-1\right)c}{d+\left(i-1\right)c}\right)\right\}+\left\{a_{i}\ln\left(\frac{b_{i}+d+\left(i-1\right)c}{d+\left(i-1\right)c}\right)\right\}\right].$$

In Figure 6d, the conductor traces on the three sides surround the 2-layer-turns of the rectangular pick-up coil on the same plane, and the magnetic flux, *ф*, generated by the electric current flowing from each side enters the coil in the same direction. Here, the magnetic-field flux incident to the coil is calculated using Equation (19). In addition, the coil output has an induction voltage twice that of a single turn coil owing to the structure of a two-turn coil. Accordingly, the mutual inductance value is also twice that for a single-layer turn.

(19) $$\phi =\frac{\mu_{o}\cdot I}{\pi }\left\{b\ln\frac{\left(a+d\right)}{d}+\frac{a}{2}\ln\frac{\left(b+d\right)}{d}\right\}$$

(20) $$M=\frac{2\mu_{o}}{\pi }\left\{b\ln\frac{\left(a+d\right)}{d}+\frac{a}{2}\ln\frac{\left(b+d\right)}{d}\right\}$$

### 3.2. Magnetic-Field Distribution

**Figure 7:**
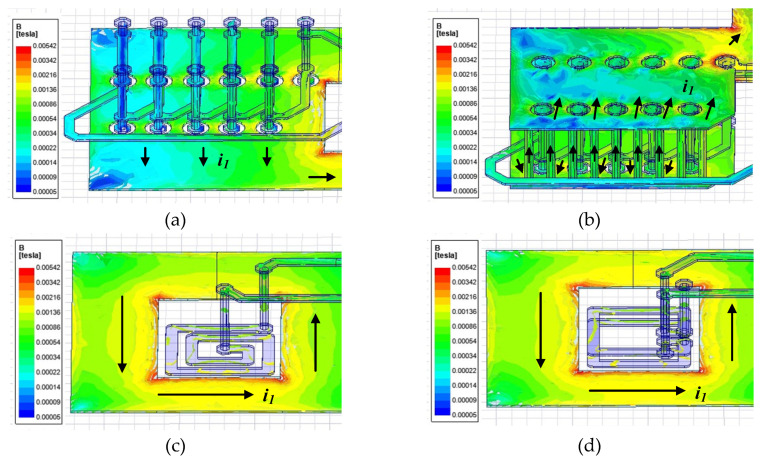
Magnetic-field flux density of (**a**) model-1; (**b**) model -2; (**c**) model-3 and (**d**) model-4 at 5-A conductor current.

Figure 7 shows the vector distribution in the magnetic-field flux between the sensor and conductor when a current of 5 A flows through the four sensor models. In the figure, the arrows indicate the direction of the magnetic flux. As shown, the model in Figure 7b with conductor traces at the top and bottom shows a denser magnetic-flux density than that of the model in Figure 7a. These sensor models have a smaller amount of magnetic-flux density entering the core than the models shown in Figure 7c,d. Table 2 shows the inductive coupling coefficient between the current trace and sensor coil by using the Maxwell- simulation tool, and that the coefficient values relative to the models in Figure 7a–d increase in size in the order of 0.143, 0.16, 0.18, and 0.21.

### 3.3. Frequency Respnse

**Figure 8:**
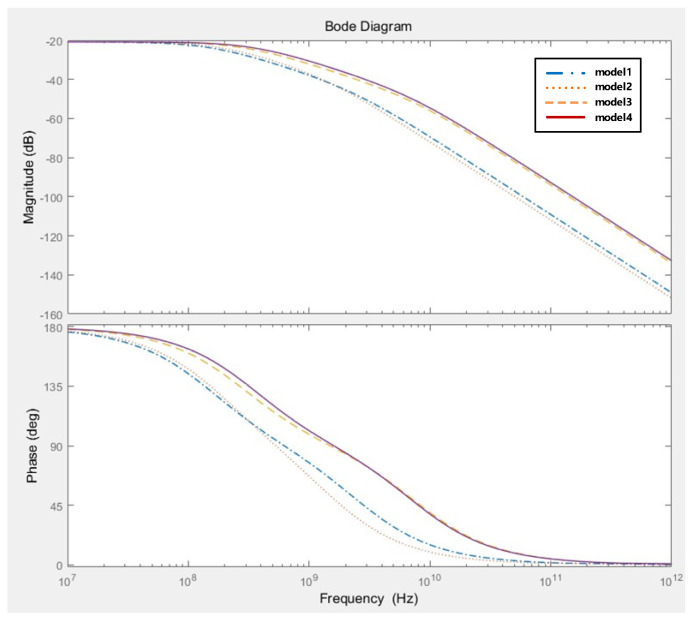
Frequency response characteristics in different coil models with the same mutual inductance value of 2.8 nH.

The GaN power semiconductor used in this study is a GaN-GS66508T(GaN systems) [44], which has a larger dv/dt characteristic than the conventional Si device. Therefore, to increase the accuracy of current detection in a transient situation, the current sensor should have a bandwidth that is 2 to 3 times larger than the device bandwidth of 150 MHz. As shown in Figure 8, for verifying the performance of a sensor, frequency response analysis of the coils with different self-inductance values was conducted based on the same mutual inductance value. That is, the curves for the four models in Figure 8 show the frequency response in the case of different self-inductance values depending on the configuration of the coils with the same mutual inductance value of 2.8 nH between the conductor and coil.

The sensor bandwidth should have a self-inductance value as small as possible under the condition that the mutual inductance value is set so as to possess sufficient measurement sensitivity due to its relationship with the coil’s self-inductance, as shown in Equation (21). According to Figure 8, models 3 and 4 show structures that receive magnetic-field flux from three sides compared to models 1 and 2, and have relatively lower turns and self-inductance values based on the same mutual inductance value of 2.8 nH. That is, a larger bandwidth can be secured based on the same sensor sensitivity.

(21) $$BW=\frac{0.35}{t_{rise}}=f_{res}-f_{c}\approx f_{res}=\frac{1}{2\pi \sqrt{L_{c}C_{c}}}$$

For the same mutual inductance value, the self-inductance values for Models 1, 2, 3 and 4 are 17.96, 15.44, 9.33, and 7.76 times the value of the mutual inductance, respectively, as shown in Table 1. That is, the 2-layer-rectangular coil model has the highest bandwidth in the standard with the same sensitivity.

## 4. Experimental Verification

**Figure 9:**
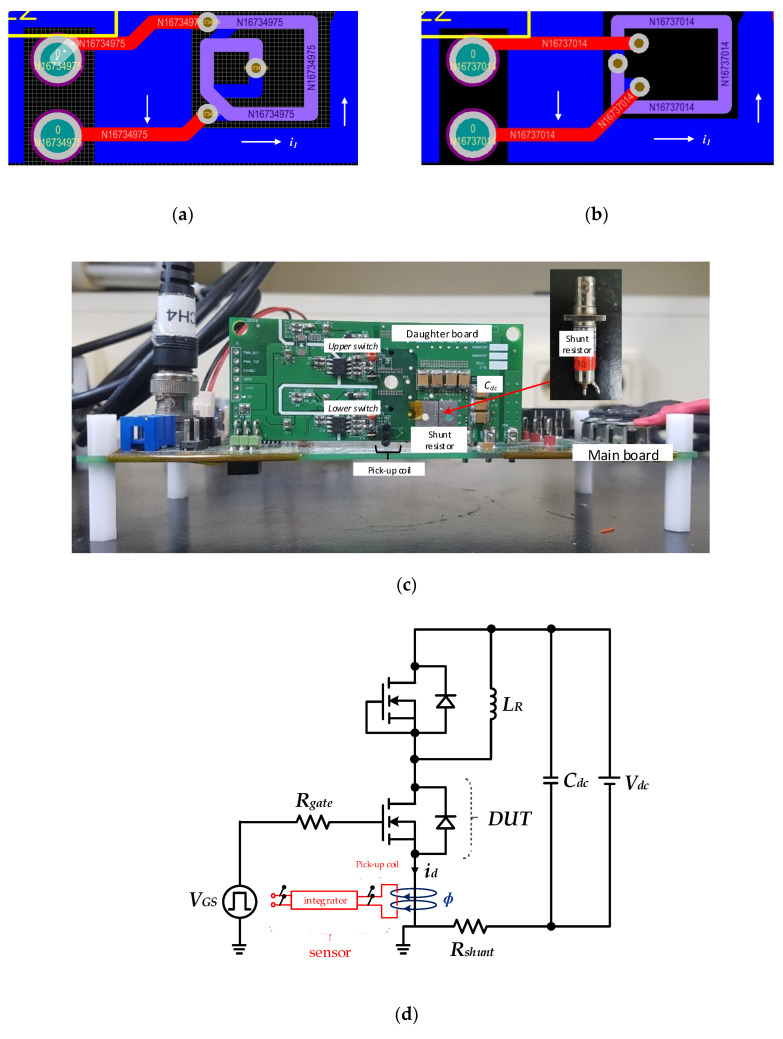
Prototype board of double pulse test: (**a**) Layout of spiral coil; (**b**) PCB layout of two-layer rectangle coil; (**c**) overall double pulse test board; (**d**) double pulse test circuit.

Figure 9a,b show the layout of the two-layer spiral coil PCB and two-layer rectangular coil in PCB proposed in this paper, as well as a close-up picture of the fabricated pick-up coil used as the prototype for the double-pulse test. Figure 9c shows the state in which the daughter and main boards with a built-in pick-up coil are vertically connected through a connector. The experiment was conducted based on the parameter values of each coil and integrator configuration of the two coils, as shown in Table 3. And the coil parameter values in Table 3 were extracted using Maxwell and Q3D tool, and the integrator parameter values were selected in consideration of the sensor’s optimal damping and bandwidth. In the case of the OP Amp constituting the integrator, LM7171 (gain bandwidth: 200 MHz) manufactured by Texas Instruments with the high slew rate of 4100 V/µs and the wide unity-gain bandwidth of 200 MHz was selected.

**Figure 10:**
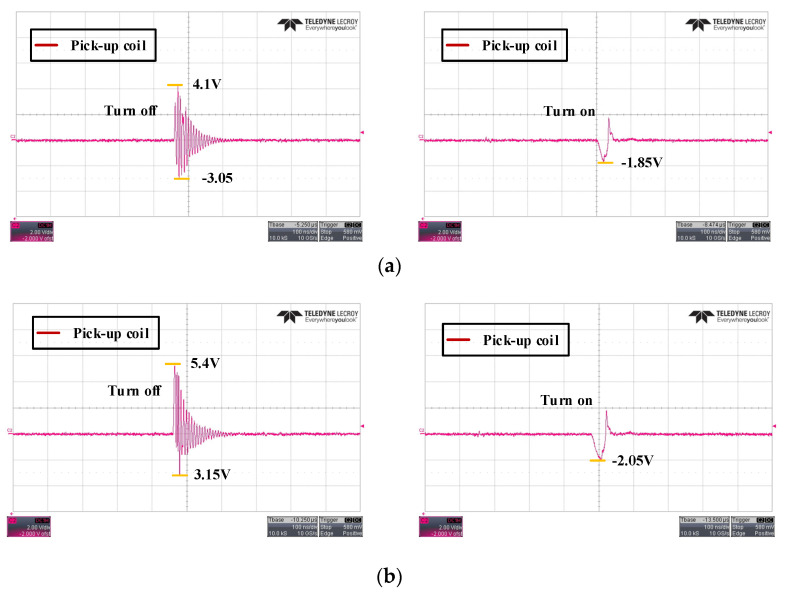
Measured coil output waveforms at 100-dc voltage: (**a**) spiral coil; (**b**) two-layer rectangular coil.

Figure 10 compares the output values of the two coils according to the switch operation in a dc voltage environment of 100 V. Figure 10a shows a positive peak value of 4.1 V during the TURN-OFF operation and a negative value of 1.85 V during the ON operation. In the case of the two-layer rectangular coil, as shown in Figure 10b, the coil with a higher mutual inductance value shows higher peak values for the ON and OFF conditions.

**Figure 11:**
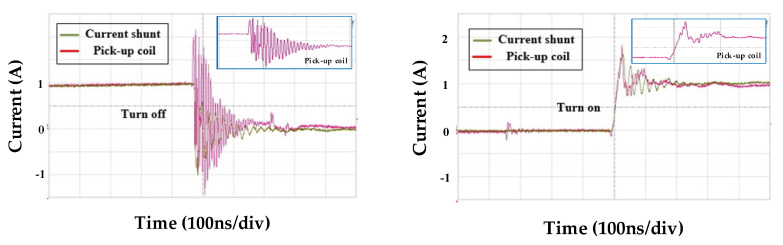
Comparison of the measured integrator output waveforms of the two-layer spiral coil and shunt resistor sensor at dc link voltage *V_dc_* of 250 V.

**Figure 12:**
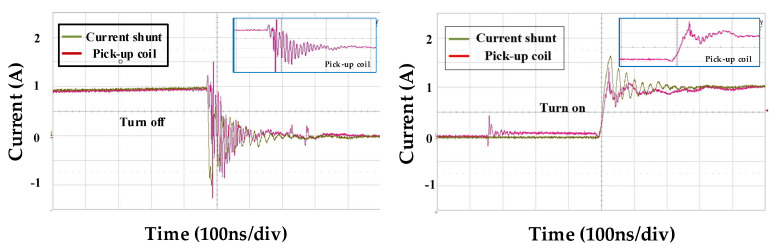
Comparison between measured integrator output waveforms of the two-layer rectangle coil integrator output and shunt resistor sensor at dc link voltage *V_dc_* of 250 V.

Figure 11 and Figure 12 provide the comparisons of the integrator output value of the two pick-up coils and the output value of the current shunt resistor sensor at 250V-dc link voltage for analyzing the switch current value during the switch operation. In this study, coaxial shunt resistor by T&M Research (SDN-141-10/2GHz bandwidth) was used for the experiment. In the experimental results of the two coil models, the switch turn-on result showed an output waveform close to that of the shunt resistance, which had a lower spike component ratio than the turn-off result. This seems to be due to the influence of coupling capacitance noise due to higher dv/dt during turn-off than turn-on.

And when comparing the performance between the two coils, the spike component was observed at a higher ratio when the switch was operated in the spiral coil compared to the two-layer rectangular coil. GaN device current measurement requires a high bandwidth, and the spiral coil has a 1.28 times lower bandwidth based on the same measurement sensor compared to the two-layer rectangular coil, so this may affect the measurement accuracy. And, to reduce the occurrence of peak values in the TURN-OFF operation of both coils, additional research is needed to reduce the influence of coupling and external noises.

## 5. Conclusions 

This paper presented a comparison between the models of pick-up coils that can be embedded in PCB circuits using the nonmodular GaN-GS66508T power semiconductors. The measurement standard of the sensor coil for measuring GaN devices must be able to secure a enough sensor bandwidth over 300 MHz and simultaneously comprise a high sensor sensitivity value. Therefore, we performed a mathematical analysis of the mutual inductances of the coil and conductor based on the magnetic-field and frequency-response analyses of the coil models to build the optimal sensor model. The analysis results showed that compared to the conventional method in which the magnetic-field flux caused by the primary current flows in the horizontal plane to the PCB layer, the spiral and two-layer rectangular structures that flow in the vertical plane could secure a larger bandwidth based on the same sensitivity sensor. In the case of sensitivity, the proposed model could secure greater sensor sensitivity based on the same bandwidth. To verify the experiment, the switching current characteristics were analyzed according to the operation of the power semiconductor by comparing the pick-up coils of the spiral and two-layer rectangular structure with a 100-mΩ coaxial shunt resistor sensor through a double pulse test.

## Figures and Tables

**Table 1:** Comparison of the dependent coil structure.

Pattern Structure	Model-1	Model-2	Model-3	Model-4
**Skeleton**	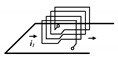	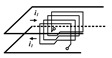	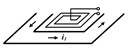	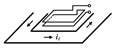
**3D-PCB Layout**	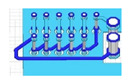	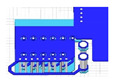	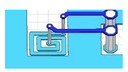	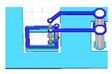
**coil mutual** **inductance** **(*M*)**	2.8 nH	2.8 nH	2.8 nH	2.8 nH
**Coil resistance** **(*R_c_*)**	0.043 Ω	0.043 Ω	0.02 Ω	0.019 Ω
**Coil capacitance** **(*C_c_*)**	1.32 pF	2.1 pF	0.45 pF	0.46 pF
**coil self** **inductance (*L_c_*)**	50.3 nH	43.2 nH	26.1 nH	21.72 nH
***L_s_*/*M***	17.96	15.44	9.33	7.76
**Bandwidth**	151 MHz	180 MHz	278 MHz	355 MHz

**Table 2:** Coil models with inductive coupling coefficient.

Sensor Structure	(a)	(b)	(c)	(d)
**Inductive Coupling Coefficient**	0.143	0.16	0.18	0.21

**Table 3:** Circuit parameters of current sensor.

Parameter	Spiral Coil	Two-Layer Rectangle Coil
Mutual Inductance	2.3 nH	3.49 nH
Self-Inductance	27 nH	21.32 nH
Self-Capacitance	0.69 pF	0.7 pF
Self-Resistance	0.034 Ω	0.034 Ω
Test current	± 10 A	± 10 A
Damping Resistance	138 Ω	120 Ω
Integrator Resistance	33 Ω	72 Ω
Integrator Capacitance	470 pF	470 pF
